# Considerations on the Use of Exogenous Fibrolytic Enzymes to Improve Forage Utilization

**DOI:** 10.1155/2014/247437

**Published:** 2014-10-14

**Authors:** Germán D. Mendoza, Octavio Loera-Corral, Fernando X. Plata-Pérez, Pedro A. Hernández-García, Mónica Ramírez-Mella

**Affiliations:** ^1^Unidad Xochimilco, Universidad Autónoma Metropolitana, Calzada del Hueso No. 1100, Colonia Villa Quietud, 04960 Delegación Coyoacán, DF, Mexico; ^2^Unidad Iztapalapa, Universidad Autónoma Metropolitana, Avenida San Rafael Atlixco No. 186, Colonia Vicentina, 09340 Delegación Iztapalapa, DF, Mexico; ^3^Centro Universitario UAEM Amecameca, Universidad Autónoma del Estado de México, Carretera Amecameca, Ayapango km. 2.5, 56900 Amecameca de Juárez, MEX, Mexico

## Abstract

Digestion of cell wall fractions of forage in the rumen is incomplete due to the complex links which limit their degradation. It is therefore necessary to find options to optimize the use of forages in ruminant production systems. One alternative is to use exogenous enzymes. Exogenous fibrolytic enzymes are of fungal or bacterial origin and increase nutrient availability from the cell wall, which consists of three fractions in different proportions depending on the species of forage: digestible, potentially digestible, and indigestible. The response to addition of exogenous enzymes varies with the type of forage; many researchers infer that there are enzyme-forage interactions but fail to explain the biological mechanism. We hypothesize that the response is related to the proportion of the potentially digestible fraction. The exogenous enzyme activity depends on several factors but if the general conditions for enzyme action are available, the potentially digestible fraction may determine the magnitude of the response. Results of experiments with exogenous fibrolytic enzymes in domestic ruminants are inconsistent. This, coupled with their high cost, has made their use unattractive to farmers. Development of cheaper products exploring other microorganisms with fibrolytic activity, such as *Fomes fomentarius* or *Cellulomonas flavigena*, is required.

## 1. Introduction

Forages are a major source of energy for ruminants [1] because cellulose, one of the main components, is the most abundant biopolymer on Earth [2]. Many forage species are of low quality because of poor digestibility and limited energy available to the animal, which contributes to a large excretion of nutrients [3] and incomplete use of fractions of the cell wall in the rumen due to the complex links which limit the degradation of nutritional compounds. This necessitates finding ways to optimize the use of forages. One option is the use of exogenous enzymes to assist with digestion [1]. The exogenous enzymes used in ruminants are from fungal (largely* Trichoderma longibrachiatum*,* Aspergillus niger*, and* A. oryzae*) and bacterial (*Bacillus* spp.,* Penicillium funiculosum*) sources with high cellulosic and hemicellulosic activity, which are incorporated in liquid or granular form with the total mixed ration, hay, silages, concentrates, supplements or premix, and increase the availability of nutrients in the cell wall [4].

In the literature there are numerous reports of evaluations of commercial and experimental enzymatic products for animals. Xylanases and cellulases have been most commonly used for ruminants, although there have been assessments with ferulic acid esterase, proteases, phytases, and amylases to break ferulic acid bridges, attack cell wall nitrogen-containing compounds, increase phosphorus absorption, and improve starch digestion, respectively [5, 6]. Responses vary with the type of forage; many researchers conclude that there is a forage-enzyme interaction but fail to present a biological explanation or hypotheses for the result [7–9]. As for any enzyme, activity depends on several factors: the type of enzyme, the species of animal to be used, pH, temperature and gastrointestinal conditions (e.g., the aqueous nature of the rumen is different from the chyme in intestine), dosage, substrate, degradation of the exogenous enzyme along the tract (rumen, stomach acid, and inhibitors), practical management conditions (stability and method of application), and other factors [10]. If conditions allow the exogenous enzyme activity, forage quality can be a determinant in the magnitude of the response to the addition of these additives. The objective of this paper is to review basic aspects of the degradation of cell wall components (cellulose and hemicellulose) to understand the response to the addition of enzymes based on forage quality. We consider the response of the enzymes as a function of the cell wall components in their three fractions: digestible, potentially digestible, and indigestible [11] and the kinetics of degradation in the rumen to elucidate why forages varying in quality and differing in digestibility fractions show differential response to the addition of exogenous enzymes.

## 2. Structure of Cell Walls

Unlike animals, plants do not have a skeletal support system to resist the force of gravity; instead, they make use of the force that builds up in cell walls which can be strong enough to hold a tree but at the same time allows flexion and compression in some plants, for example, when there is wind [12]. Plant cell walls are composed of cellulose (35–50%), hemicellulose (20–35%), and lignin (10–15%).

Cellulose is a linear molecule of insoluble *β*-glucans, comprising more than 15 000 D-anhydroglucopyranose residues linked by *β* (1-4) bonds [2]. Cellulose is found almost exclusively in the cell walls of plants and trees, but it also occurs in various living organisms such as tunicates and algae and is also produced by several species of bacteria as exopolysaccharide membranes [13, 14]. However, the cellulose produced by microorganisms usually has different properties than that produced by plants, so their applications are also different [15]. Plant cellulose is hydrolysed by various cellulases: endoglucanases hydrolyse cellulose chains randomly and produce oligomers of cellulose; exoglucanases produce cellobiose by hydrolysing cellulose chains in their final nonreducing linkage, and *β*-glucosidases release glucose from cellobiose [4]. Experimentally, purified cellulose has been evaluated in several studies of hydrolysis and microbial utilization. However, cellulosic biomass is more complex than purified cellulose because it forms a complex with hemicellulose and lignin. Moreover, there are differences between tissues of plants; for example, mesophilic tissue has thinner and less lignified cell walls which are readily degraded by fibrolytic enzymes while sclerenchyma is highly lignified and has thicker walls [13].

Hemicellulose is a heterogeneous group of polysaccharides characterised by *β* (1-4) linkages in an equatorial configuration, within which are included xyloglucans and glucuronoxylans (present in dicots), glucuronoarabinoxylans (in grasses and conifers), glucomannans (dicots and grasses), and galactoglucomannans (conifers) [16]. Xylan is the main component of the hemicellulose which is, after cellulose, the most abundant polysaccharide in nature, constituting 30–35% of the cell wall of cereals and grasses. Hemicellulose is considered an important fraction in ruminant nutrition [2]. Hemicellulose is synthesized in the Golgi apparatus of plant cells by the action of several glycosyltransferases found between the *β*-(1-4)-glucan synthase, *β*-(1-4)-xylan synthase, and *β*-(1-4)-mannan synthase [16].

Lignin is a branched polymer formed by four alcohols (coniferyl, hydroxyconiferyl, coumaryl, and sinapyl alcohols) from the phenylpropanoid pathway of plants, resulting in different forms of lignin: guaiacyl, 5-hydroxygualacyl,* p*-hydroxyphenyl, and syringyl lignin deposited in cell walls as part of the maturation process of the plants after completion of cell growth [17]. The proportion of these phenolic compounds varies depending on the nature of the plant species, organ, and cell wall layers [18]. Guaiacyl lignin represents 95% of the lignin found in gymnosperms, while there are large amounts of both syringyl and guaiacyl lignin types in angiosperms. The hydroxyphenyl lignin type is found in most plants in small amounts, but the hydroxyguaiacyl type is only found in one variety of corn (Bm3). Lignin biosynthesis begins with phenylalanine. The second precursor is tyrosine which acts through tyrosine ammonia lyase to form coumaric acid. Other enzymes involved in the synthesis of lignin are phenylalanine ammonia lyase, cinnamate 4-hydroxylase, 4-coumaroyl hydroxylase, 0-methyltransferase, ferulate 5-hydroxylase, 4-coumarate-CoA ligase, 4-coumaroyl-CoA hydroxylase, caffeoyl-CoA 0-methyltransferase, cinnamoyl-CoA reductase, cinnamyl alcohol dehydrogenase, and peroxidase [17]. Lignin has a high molecular weight which gives stiffness to the cell wall of the plant, limiting the availability of the structural carbohydrates to rumen microorganisms [19]. This, in turn, limits its digestibility and therefore overall forage nutrient availability [20].

In addition to their structural role, cell walls are a source of energy for the plant. For example, seeds of many cereals such as corn, wheat, and rice contain an endosperm consisting predominantly of starch, but the cell wall of the pericarp serves as an energy source during germination; it has been estimated that up to 18% of the glucose of germinated barley grains is released from (1,3;1,4)-*β*-D-glucans from the cell walls [12].

## 3. Fractions of Neutral Detergent Fiber as a Function of Digestion Kinetics

The neutral detergent fiber (NDF) contains three fractions classified according to the kinetics of digestion in the rumen: digestible, potentially digestible, and indigestible. The potentially digestible fraction disappears from the rumen by passage and digestion. The indigestible fraction does not supply nutrients to the ruminant, passing undigested to appear in the feces [11]. This adversely affects the digestibility of organic matter [21]. The indigestible fraction can be estimated with incubations* in situ* or* in vitro* for more than 96 hours [22]. Theoretically,* in situ* incubations provide more useful results because digestion occurs directly in the rumen of the animal with the use of nylon bags; however, this method has disadvantages. One is that the pore size of the bag is critical: small pore size limits the entry of some ruminal protozoa but larger pore sizes allow forage losses or entry of ruminal digesta, so the risk of underestimating or overestimating forage digestibility is high [23, 24]. Nousiainen et al. [21] reported that the indigestible NDF fraction of grass silage was 8.7% of the dry matter (DM) incubated* in situ* for 12 days (to ensure complete digestion) in bags with pores less than 20 *μ*m (to avoid losses). In fresh and hayed forages, the indigestible fraction varies depending on the species. For example,* Lolium perenne* had 12.8% and* Dactylis glomerata *had 28.9%;* Paspalum notatum* ranged from 21.0 to 33.0%;* Megathyrsus maximus* had 35.2%; oat straw (*Avena sativa*) varied between 21 and 36%;* Cynodon dactylon* had 43.9%; wheat straw (*Triticum* ssp.) changed from 39.6 to 49.0%; and sugar cane bagasse (*Saccharum officinarum*) had 76.8% [19, 25, 26]. In general, lower quality forages, such as straws or bagasse, have a higher indigestible fraction. We hypothesized that the addition of exogenous enzymes will have less of an effect than in higher quality forages with lower proportions of indigestible fractions. This would explain why experiments using higher quality forages result in a positive response in digestion to fibrolytic enzymes [9]. However these NDF fractions have been not well evaluated in studies investigating addition of enzymes to forage. In this regard, Nadeau et al. [27] reported that the concentration after* in situ* digestion of potentially digestible NDF, cellulose, and hemicellulose from silages of alfalfa (*Medicago sativa*) and orchardgrass (*Dactylis glomerata*) was, in average, 33, 37, and 27% lower, respectively, when treated with an additive enzyme (extracted from* Trichoderma longibrachiatum*) than without it.

The indigestible NDF fraction is related to lignin [28] and the amount of lignin is negatively related to forage digestibility [17, 29]. Because the process of lignification is genetically regulated, there are differences between plant species and between genotypes of the same species [19]. Nadeau et al. [27] reported that 60 days after ensiling with enzyme additive, the concentration of NDF decreased 23 and 15% in orchard grass (*Dactylis glomerata*) and alfalfa (*Medicago sativa*), respectively, compared with control treatment. This is attributed to the greater amount of lignin in the alfalfa. To improve its digestibility, genetically modified forages have been developed [30]. Alfalfa is an angiosperm that contains mostly guaiacyl and syringyl lignin. Reducing the expression of genes encoding for caffeic acid 3-0-methyltransferase enzyme decreased lignin content, completely losing syringyl lignin (which increases with maturity of the forage), and decreased caffeoyl-CoA 3-0-methyltransferase, reducing the amount of lignin guaiacyl. Thus, it has been possible to increase the* in vitro* and* in situ* alfalfa true digestibility by 1–5% and 2.8–6%, respectively, by decreasing the lignin content by up to 50% in alfalfa lines with less caffeoyl-CoA 3-0-methyltransferase enzymatic activity. Meanwhile, Baucher et al. [31] developed several lines of genetically modified alfalfa to diminish the activity of the cinnamyl alcohol dehydrogenase, an enzyme that catalyses the last step of the synthesis of lignin in plants, so that some lines reduced to 16% and 56% the amount of guaiacyl and syringyl lignin, respectively, allowing an increase of 15 to 23% on* in situ* DM digestibility.

Other factors influencing the NDF digestibility of forages are lignin bonds with other carbohydrates, creating a barrier which prevents microorganism enzymes from acting on them [17], and which are resistant to acid and alkaline hydrolysis in the gastrointestinal tract [32]. In addition to forage maturity, the number of cuts, the latitude, climate [33], morphological characteristics of the plant, and the crystallinity of the cellulose [22, 34] affect forage digestibility.

## 4. Use of Fibrolytic Enzymes in Ruminants

Fibrolytic enzymes (*β*-glucanases and xylanases) were initially used only for pigs and poultry [35–39] in order to remove some antinutritional factors and to degrade the pericarp covering the endosperm of the grain. The pericarp is composed of *β*-glucans, xylan, and cellulose, indigestible compounds for nonruminants [40, 41]. Fibrolytic enzymes were not used in ruminants because it was thought that they would be rapidly destroyed by rumen proteases, and also because ruminal microorganisms can degrade fibrous substrates [3]. However, the ruminal digestibility of NDF is rarely greater than 50%, less when rumen conditions are not favorable for adequate fibrolytic activity, as occurs with high grain diets [42]. Therefore, the addition of enzymes in ruminant diets could increase digestibility of fibrous feeds, decreasing feeding costs by reducing the use of grains, widely used in rations, and thus improving productivity and feed conversion [5]. Several studies show that exogenous fibrolytic enzymes increase the digestibility of DM, NDF, and acid detergent fibre (ADF) [43–45].

Most studies exploring the use of exogenous enzymes for dairy cattle have focused predominantly on diets of high quality forages (e.g., alfalfa and corn silage [46]) which have shown positive results [7]. Oba and Allen [47] indicate that, for each unit increase in* in vitro* digestibility of NDF, DM intake and 4% fat-corrected milk production increased by 0.17 kg/d and 0.25 kg/d, respectively. Gado et al. [48] reported 13% greater DM intake and 23% greater milk production in cattle fed with fibrolytic enzymes compared with the control group. However, some studies showed no change in either DM intake or production [49–52], and others reported no changes in milk production but decreases in DM intake [53, 54].

An important aspect of the use of exogenous enzymes in ruminant feeding is the potential to reduce the grain level in the ration [55] reducing costs. Some evaluations in Mexico with dairy cattle (for 114 days) show an increase in production of 1.5 kg/d, but the cost of the enzymes was US$0.39/dose/cow, leaving a profit of US$0.09/cow/day. Economic losses could be substantial if the investment in enzymes is not matched by increases in production because lower forage quality limits the effect of the enzyme. This may present a limitation on the use of enzymatic additives because they represent an increase in the cost of production [56].

It has been reported that the use of exogenous fibrolytic enzymes improves the energy status of cows by reducing plasma concentrations of *β*-hydroxybutyrate, indicating that the mobilization of fat from adipose tissue is reduced both in early [54] and in middle lactation [52]. In contrast, there is evidence that exogenous enzymes increase microbial protein synthesis, an indicator that the bacterial population of the rumen is increased [50]. However, other studies show no changes in the microbiota [57, 58]. Results from these experiments indicate that the use of enzymes can promote a greater flow of carbon for volatile fatty acids and/or microbial protein synthesis, but there is a need to clarify under what conditions the synthesis of microbial protein is favoured.

In beef cattle, results are more inconsistent. Salem et al. [45] reported increases of 16% in weight gain and 9% improvement in feed conversion; however, Eun et al. [59], Ware et al. [60], and Krueger et al. [61] reported that supplementation with exogenous enzymes did not affect weight gain in steers as in other studies [51, 60], although some studies found increases in DM intake [61]. The inclusion of fibrolytic enzymes in finishing diets containing low amounts of forage (~8–12%) should not have an impact on the productivity of cattle; however, as a result of high concentration of starch in these diets, ruminal pH is low (<6) for long periods during the day, affecting the ability of microorganisms to degrade fibre. Therefore the use of fibrolytic exogenous enzymes in high grain diets could improve the digestibility of the forage fraction. Krause et al. [62] reported a 28% increase in ADF digestibility of a diet with 95% concentrate (mostly barley grain) in finishing diets for bulls supplemented with a commercial enzyme; Beauchemin et al. [63] reported that fibrolytic enzymes improve feed conversion by 11% only when the diet contained barley grain, but not with corn grain, suggesting that the enzymes could be acting on the pericarp (husk).

The high cost of enzyme products (compared to ionophores, implants, and antibiotics) has limited its use in feedlot conditions [3]. Nevertheless, exogenous fibrolytic enzymes can improve carcass characteristics; Eun et al. [59] mention that the fat thickness at the 12th rib was reduced (8.8%) in cattle supplemented with a commercial enzyme additive, while Vargas et al. [64] report that hot carcass yield was improved (6.7%) and the cutting force was reduced (11.4%). Further studies are required to confirm these results and to assess whether these changes in carcass quality offset the cost of the enzymes.

The use of fibrolytic enzymes in grazing cattle in the tropics is not common. Nevertheless, there is experimental evidence from* in vitro* studies showing that sugarcane digestibility and microbial protein synthesis can be improved [65]. Gómez et al. [66] indicate that increasing enzyme dosage from 15 to 30 g/day increased weight gain by 65% due to greater digestibility of tropical grasses and sugar cane as a supplementary forage. However, the economic analysis of the two experiments (80-day trial) showed that the dose of 30 g/day is not profitable, but a dose of 15 g/day allows a profit of US$0.1–0.17/steer/day with a daily investment of US$0.59/steer/day [56]. Some studies at feedlots where straw is used as forage showed positive responses at very high doses [67] that are not profitable, whereas in other studies there was no response even at high doses [64].

In the case of fattening lambs, there are several reports where there was positive response in weight gain by the addition of fibrolytic enzymes [68–70]. In another studies, there was no response [71, 72]. In one experiment where there was positive response [68], one enzyme resulted in a profit of US$0.11/lamb/day after an investment of US$0.04/animal/day while the other commercial enzyme resulted in losses of US$0.22/lamb/day. In an experiment where there was no response to the enzymes [71], the estimated daily losses were US$0.33/lamb/day [56].

## 5. Fibrolytic Enzymes and Fermentation Gases

The use of fibrolytic enzymes in ruminants can also be related to greenhouse gas emissions. Globally, livestock contributes 18% of emissions of greenhouse gases. In 2005, countries of Europe, North America, and those related to the former Soviet Union emitted 25.5% of methane (CH_4_), while Asia, Africa, and Latin America accounted for 69% of enteric emissions [73]. Usually the most productive animals consume more food, produce more excrement, and emit higher absolute amounts of greenhouse gases than less productive animals. However, when expressed per unit of animal product, the most productive animals generated significantly fewer greenhouse gases than less productive animals [74]. Flachowsky [75] estimated that a cow producing 40 kg/day of milk emits 50% less carbon dioxide (CO_2_) per kilogram of milk produced than a cow producing 10 kg/day, while a calf gaining weight at 1.5 kg/day emits 70% less CO_2_ than one gaining 1.0 kg/day. One way to increase animal productivity is to improve the quality of feed. In many developing countries it is common for ruminants to be fed with crop residues or low quality pastures. In this case, exogenous enzymes could be an option to improve digestibility [76]. Arriola et al. [53] reported reduced production of methane (<11%) in cows on diets with 48% concentrate supplemented with fibrolytic enzymes, but not when the diet contained only 33% concentrate, indicating that the response is a function of the forage : concentrate ratio.

Several reports indicating that the use of enzymes increases the* in vitro* gas production should be interpreted carefully. Tang et al. [77] reported increases in* in vitro* gas production using various highly fibrous substrates (corn stover, rice straw, and wheat) because of the effect of fibrolytic enzymes, but they did not measure CH_4_ production. Soltan et al. [78] indicated that the use of exogenous enzymes, particularly cellulases, increases unmodified CH_4_ production, indicating that there is a forage-enzyme interaction, but they did not provide a biological explanation. The increase in gas production by the action of exogenous enzymes can be attributed to increased degradation of substrates in the rumen. Note, however, that previous studies did not determine microbial nitrogen and it is important to register the carbon flow in volatile fatty acids (VFAs), gas, and bacterial biomass. Leng [79] pointed out that improving the conditions of the rumen increases bacterial synthesis (and thus the amount of microbial protein available to ruminants) and decreases the production of CH_4_. This finding is consistent with Gado et al. [48] who found that the use of exogenous enzymes increased the amount of rumen bacteria because the fiber digestibility was improved. However, there are too few studies on fibrolytic enzymes that measure VFAs, CH_4_ production, and microbial protein synthesis to reach a conclusion on the effect of enzymes in the production of greenhouse gases [80].

In contrast, there are studies reporting no changes in* in vitro* gas production [57, 58] by exogenous enzymes. Kung Jr. et al. [81] reported that gas production and VFAs were not affected whereas Yang et al. [82] found that the final concentration of VFAs increased by almost 9% with the enzymes and that the lag phase was reduced with concentrates (barley-based), suggesting that the enzymes improve conditions for the beginning of digestion. Inconsistent results indicate that further studies are required to demonstrate the effects of exogenous fibrolytic enzymes on* in vitro* kinetic parameters including microbial protein synthesis.

## 6. Mechanism of Action

The increase in milk and meat production by the effect of exogenous enzymes is due primarily to the increase in fiber digestion of feed components (NDF and ADF). This result has been found in studies both* in vitro* [46, 50, 83] and* in vivo* [45, 48, 61]. The effect is greater when enzymes are applied to the feed just before it is consumed [3]. Beauchemin et al. [3] postulated that it is unclear whether the effect of enzymes is the result of their action in the feed or in the rumen. However the conditions of pH, temperature, and contact substrate outside the rumen are not conducive to the action of exogenous enzymes. Therefore their effect must be because of their action inside the rumen. In addition, synergy among different enzymes has been reported raising the possibility of combining different enzymes from various microorganisms to develop products with higher activity. For example, Yang et al. [83] reported higher digestibility of DM* in vitro* when two enzymatic products were applied together, although this effect was only apparent using alfalfa hay (better quality forage). Neither enzyme in isolation nor both in synergy had any effect when used with rice straw (lower quality forage). We hypothesize that the lack of response using straw is because it has a lower potentially digestible fraction. This is an indication that if conditions permit the action of the enzyme, forage quality factors can determine the response to the addition of these additives.

Substrate conditions affect the action of exogenous enzymes. Exogenous enzymes act more effectively in wet feed than in dry feed because water facilitates the dissemination of the enzymes and is essential for hydrolyzing fiber polymers to release the monomers [3]. The activity of the hemicellulolytic exogenous enzymes may be reduced when used with silage, possibly due to the presence of characteristics of fermented feed which decrease the activity of enzyme *β*-(1-4)-xylanase up to 50%. In contrast, the activity of cellulolytic enzymes is not affected [84].

## 7. Enzymatic Activity

The types of cellulases and hemicellulases and their activity differ among different commercial products currently available, directly affecting the products' ability to degrade the cell wall components of forages [4]. Enzymatic activity depends on several factors including the microorganism of origin (fungal or bacterial), the type and stability of the enzyme, the type of forage and livestock animal to be used, the pH, temperature and conditions of solution in the gastrointestinal tract, the dose, substrate, enzyme degradation in tract (rumen, stomach acid, and inhibitors), and handling conditions of the product including application method [10, 85]. For example, Márquez-Araque et al. [86] reported that* Fomes* sp. EUM1 (formerly classified as* Trametes* sp.) has 5 and 7 times more xylanolytic activity and 10 and 8 times more cellulolytic activity than* Aspergillus niger* and* Pleurotus ostreatus*, respectively. Ramírez et al. [87] examined the activity of three commercial enzymes under different pH conditions, and all of them showed higher activity at pH 6.5 than at pH 5.5. Arce-Cervantes et al. [6] found that the xylanases from* Fomes* sp. EUM1 increased 136% when 20% wheat bran was added to the substrate (corn stover), while cellulolytic activity was not affected. Therefore, culture conditions affect the levels and type of occurring enzymes, which in turn will affect the activity of enzymes.

Due to its heterogeneity, the cell wall cannot be used as substrate to assess the enzymatic activity; instead purified substrates are used: carboxymethylcellulose for endo-*β*-(1-4)-glucanases, avicel for exoglucanases, and xylan (oat or birchwood) for xylanases [4]. In order to evaluate an enzyme or a mixture of commercial enzymes, it is important to express the enzymatic activity in International Units (IU), where an IU is defined as the amount of enzyme required to release 1 mg of reducing sugars (xylose and glucose). It is also necessary to know the stability or enzyme resistance to degradation in rumen conditions [85]. Some commercial enzymes are glycosylated, which prevents degradation of rumen microorganisms. This, in turn, explains the positive effects* in vitro* and* in vivo* [8, 88–90]. It is important to compare the activity of commercial products with those produced by rumen microorganisms to ascertain their potential effects. For example, the cellulolytic activity in* Fomes* sp. is 40 to 50 times higher and the xylanolytic activity up to 200 times higher than ruminal microbes [86, 87].

## 8. Development of New Enzymes

One option to promote the use of enzymes in ruminant production systems is to develop cheaper products. Development should consider the use of cheaper materials that are widely available at any time of year and nontoxic. It may be necessary to remove some unwanted constituents, such as proteases, using boron compounds with glycerol and propylene glycol, and to use some stabilizers (sodium chloride, glycerol, and propylene glycol) and preservatives (sodium benzoate and potassium sorbate), particularly in liquid preparations. Powdered preparations can be “protected” to control dust and to maintain enzyme activity. Additional factors to consider are the development of thermostable enzymes to resist the temperature reached by the pelleting process and development of enzymes able to resist natural inhibitors from grains [2]. These factors will increase the cost of the product. Moreover, it is important to develop and test enzymes in conditions similar to the rumen (pH 6.0 to 6.7 and 39°C). Sometimes, enzyme products are tested at 60°C in acidic pH (pH 4 and 5) which overestimates their activity in the rumen [4, 85]. Most of the* in vivo* studies in the literature are conducted with expensive commercial enzymes which do not always show positive results increasing milk or meat production; however, a wide variety of bacteria and fungi with potential for producing enzymes have not yet been explored with animals. Some organisms with fibrolytic capacity that could contribute to technological development of new products are the fungi* Fomes fomentarius, Trametes versicolor, Bjerkandera adusta, Pleurotus ostreatus, Fomes* sp. EUM1, and* Agaricus bisporus* [86, 91, 92] or the bacterium* Cellulomonas flavigena* [93]. In some cases commercial preparations of fibrolytic enzymes may vary between batches, which could explain part of the variability in the response. It would be advisable to test stability and quality in these types of studies [85].

In conclusion, the response to exogenous enzymes can be modified by forage quality, in particular the proportion of the potentially digestible fraction. This requires studies forage-enzyme characterization including kinetic studies of cell wall digestion. Commercial exogenous enzymes are an option to improve digestion of nutrients and productivity of ruminants, but due to costs, their use is only recommended with high quality forage. It is important to promote the development of new low-cost enzymatic products that are profitable for incorporation in various ruminant production systems.
